# Supplementary material for: Dietary Behaviors, Sugar Intake, and Public Awareness of Nutritional Labeling Among Young Adults: Implications for Oral and Systemic Health
Source: Nutrients. 2025 Dec 27;18(1):91. doi: 10.3390/nu18010091 (PMC12787495; doi:10.3390/nu18010091)
Supplement: Supplementary file 1 [file nutrients-18-00091-s001.zip › nutrients-4048810-supplementary.pdf]

**Questionnaire on Simple Carbohydrate Consumption and Food Labeling**

**Sociodemographic Data**

**1. What is your age?**
☐ 18–21 years
☐ 22–25 years
☐ 26–30 years

**2. Gender:**
☐ Female
☐ Male

**3. Place of residence:**
☐ Rural
☐ Urban

**4. What is the highest level of education you have completed?**
☐ Primary school
☐ High school
☐ University studies
☐ Postgraduate studies

---

**Dietary Knowledge and Behaviors**

**5. How important do you consider the consumption of simple carbohydrates in your daily diet?**
☐ Very important
☐ Important
☐ Moderately important
☐ Slightly important
☐ Not important at all

**6. How often do you read the nutritional values on food product labels?**
☐ Very often
☐ Often
☐ Rarely
☐ Never

**7. In which form do you most frequently consume simple carbohydrates?**
☐ Dairy products

☐ Whole grains
☐ Sweets
☐ Carbonated beverages

**8. For which of the following products do you most frequently read the label?**
☐ Dairy products
☐ Whole grains
☐ Sweets
☐ Carbonated beverages

**9. Which information do you pay attention to when reading the label of a product containing simple carbohydrates?**
☐ Expiration date
☐ Ingredient list
☐ Nutritional information
☐ Serving size recommendation
☐ Allergen declaration

**10. From which sources do you obtain information regarding simple carbohydrate consumption?**
☐ Books
☐ Magazines
☐ Internet
☐ Television
☐ I do not seek information

**11. How informed are you about the classification of simple carbohydrates into beneficial and harmful types?**
☐ Very informed
☐ Informed
☐ Moderately informed
☐ Slightly informed
☐ Not informed at all

**12. How often do you consume sweets and carbonated beverages?**
☐ Very often
☐ Often
☐ Quite rarely
☐ Never

**Attitudes and Perceptions**

**13. Do you agree with the statement “Food labeling is truthful”?**

☐ Totally disagree
☐ Disagree
☐ Neither agree nor disagree
☐ Agree
☐ Totally agree

**14. How important do you consider awareness of the amount of sugar in foods?**

☐ Very important
☐ Important
☐ Moderately important
☐ Slightly important
☐ Not important at all

**15. How often do you usually visit the dentist?**

☐ Once every 6 months
☐ Once a year
☐ Once every few years
☐ Only in emergency situations
☐ Never

**16. How informed are you about the harmful effects of simple carbohydrates on oral health?**

☐ Very informed
☐ Informed
☐ Moderately informed
☐ Not informed at all

**17. Have you associated the occurrence of dental caries with excessive simple carbohydrate consumption?**

☐ Yes
☐ No

**18. Are you willing to reduce your simple carbohydrate intake to improve oral health?**

☐ Extremely unlikely
☐ Unlikely
☐ Neutral
☐ Likely
☐ Extremely likely

**19. Which factors influence your choice of products containing simple carbohydrates?**

☐ Taste

☐ Nutritional value

☐ Price

☐ Brand

**20. To what extent do you agree with the statement "Taste is one of the main factors influencing the purchase of a food product"?**

☐ Totally disagree

☐ Disagree

☐ Neither agree nor disagree

☐ Agree

☐ Totally agree
